# Assessment of Zn and Cu in piglets’ liver and kidney: impact in fecal *Enterococcus* spp.?

**DOI:** 10.1007/s11356-024-32495-8

**Published:** 2024-02-21

**Authors:** Maria M. Donato, Gabriela Assis, Olga Cardoso, Bárbara Oliveiros, Andreia Freitas, Fernando Ramos

**Affiliations:** 1 Faculdade de Medicina, Universidade de Coimbra, CIMAGO, Azinhaga de Santa Comba, 3000-548 Coimbra, Portugal; 2 Laboratório de Controlo da Alimentação Animal, Unidade Estratégica de Investigação E Serviços, Tecnologia E Segurança Alimentar, Instituto Nacional de Investigação Agrária E Veterinária, I.P., Av. da República, Quinta Do Marquês, 2780-157 Oeiras, Portugal; 3 Faculdade de Farmácia, Universidade de Coimbra, CERES, Azinhaga de Santa Comba, 3000-548 Coimbra, Portugal; 4 Faculdade de Medicina, Universidade de Coimbra, LBIM, Azinhaga de Santa Comba, 3000-548 Coimbra, Portugal; 5 Faculdade de Medicina, Universidade de Coimbra, CIMAGO, I-CBR, Azinhaga de Santa Comba, 3000-548 Coimbra, Portugal; 6 Laboratório Nacional de Referência Para a Segurança Alimentar, Instituto Nacional de Investigação Agrária E Veterinária, I.P., Rua Dos Lágidos, Lugar da Madalena, 4485-655 Vairão, Vila Do Conde Portugal; 7 REQUIMTE/LAQV, Rua Dom Manuel II, Apartado 55142, 4051-401 Porto, Portugal; 8 Faculdade de Farmácia, Universidade de Coimbra, Azinhaga de Santa Comba, 3000-548 Coimbra, Portugal

**Keywords:** *Enterococcus*, Piglets, Zinc, Copper, Metal susceptibility, Antibiotic susceptibility

## Abstract

Zinc and copper have been used as growth promotors in alternative to antibiotics in pig’s diet. The aim was the ascertainment of the Zn and Cu concentrations in piglets’ liver and kidney and their impact in the reduced susceptibility to Zn, Cu, and antibiotics in enterococci, used as microbiota biomarker. Zn and Cu were determined in the livers and kidneys of 43 piglets slaughtered in Portugal, by flame atomic absorption spectrometry. Enterococci were isolated from feces for determining the identification of species (*E. faecalis*, *E. faecium*, and *Enterococcus* spp.); susceptibility to vancomycin, ciprofloxacin, linezolid, tigecycline, ampicillin, imipenem, and metals; and Cu tolerance genes. In piglets with Zn and Cu high or toxic levels, enterococci had reduced susceptibility to ions, reinforced by the presence of Cu tolerance genes and by resistance to antibiotics. The study relevance is to show the relationship between these metals’ levels and decreased susceptibility to Cu, Zn, and antibiotics by enterococci. From the results, it could be supposed that the piglets were being fed with high doses of Zn and Cu which could select more resistant bacteria to both antibiotics and metals that could spread to environment and humans.

## Introduction

One of the most common threats to the global pig industry is post-weaning diarrhea in pigs. The European Union–wide ban on using antibiotics as growth promoters in feed came into force on 1 January 2006 as part of the Commission’s overall strategy to tackle the emergence of bacteria and other microbes resistant to antibiotics. As a result, one of the alternatives to antibiotics in weaning piglets is the supplementation of pig feed with zinc and copper. Farmed animals, such as pigs and poultry, receive additional Zn and Cu in their diets via supplementation in compound feeds to prevent diarrheal diseases and as an alternative to antibiotics for growth promotion (Rensing et al. 2018; Wales and Davies 2015).

Zn and Cu are essential trace elements for every life form and carry out many biological functions. Both Zn and Cu are cellularly ubiquitous and are important structural components or regulatory cofactors of many different enzymes in several important biochemical pathways in plants and animals. Organisms have developed homeostatic capacities that allow some control over internal concentrations of essential elements, maintaining optimal levels despite fluctuating external availability (Hill and Shannon 2019; Yazdankhah et al. 2014).

Swine tolerate high levels of dietary Cu and begin to accumulate Cu in the liver at high Cu intake levels (up to 50 times the dietary requirement). The liver is the main reservoir of Cu and the main mediator of its homeostasis in the body. Under normal exposures, retention of absorbed Cu in the body is regulated by hepatobiliary excretion, which accounts for 98% of Cu excretion, with the remainder lost via urine (FEEDAP 2016).

In general, animals can tolerate much higher levels of Zn than those naturally present in feedstuffs and/or in balanced complete/complementary diets supplemented up to the European Union maximum levels (European Parliament and Council of the European Union 2003). Above the physiological requirement, the accumulation of Zn in the liver, kidney, and intestine allows the regulation of Zn homeostasis through the scavenging of excess Zn (Food and Authority 2014).

The genus *Enterococcus* is ubiquitous and species are found in a variety of habitats including soils, sediments, freshwater, seawater, beach sand, and various plants. *Enterococcus* spp. are also common members of animal and human gastrointestinal microbiota, with concentrations in human and animal feces typically 10^3^–10^7^ cells per gram, and can withstand many environmental stressors. Zn and Cu may be toxic to enteric bacteria of livestock, which evolved mechanisms to avoid such toxicity. The development of Cu-reduced susceptibility in enterococci is associated with the presence of a Cu resistance gene (*tcr*B) located in a plasmid and the *copYZAB* operon, which encodes four proteins (CopY, CopZ, CopA, and CopB) that work together to maintain tolerable levels of Cu in the cell (Hasman et al. 2006). Zn-reduced susceptibility is often associated with efflux (Rensing et al. 2018).

The aim of the present study is to ascertain the levels of Zn and Cu in the kidney and liver of piglets slaughtered in Portugal and to determine the susceptibility to antibiotics and metals in enterococci species isolated from feces (as representative of the intestinal microbiota of piglets), in order to observe if there is a correlation between Zn and Cu concentrations in the kidney and liver and the reduced susceptibility of enterococci to Zn, Cu, and antibiotics.

## Materials and methods

### Sampling

In this study, samples of feces (*n* = 43), liver (*n* = 42), and kidney (*n* = 43) were taken from randomly selected healthy animals (*n* = 43 piglets; weighing 5–8 kg) from a slaughterhouse located in Mealhada (center of Portugal) that receives piglets from around Portugal, between October 2018 and May 2019. Sample collection was performed under supervision of a veterinarian. The 43 piglets came from 10 different farms. Nine piglets were from Aveiro and were collected three times (four in December; three in April; two in May); nine were from Pombal and were collected three times (two in November; four in March; three in April); eight were from Faro and were collected twice (four in March; four in May); and the rest were from Portela do Outeiro (five in March), Alcobaça (four in February), Batalha (one in April), Caldas da Rainha (three in April), Mação (two in October), Mira (one in December), and Castelo Branco (one in May).

Samples collected from each animal consisted of a minimum of 10 g of feces; 200 g of liver (right lobe); and a whole kidney. Each sample was stored in a plastic bag and immediately transported to the laboratory. Liver and kidney samples were stored at – 18 °C until analysis. Processing for the isolation of *Enterococcus* species in the feces was performed on the same day.

### Isolation of *Enterococcus* spp

Feces (10 g) were mixed with 90 mL of a tryptone salt broth (Oxoid). Suspensions were transferred to sterile stomacher bags and homogenized for 2 min in a stomacher blender. Serial dilutions were made in tryptone salt broth. Dilutions were inoculated in Slanetz-Bartley Agar (Oxoid) (37 °C, 24–48 h). Up to four red-colored colonies were selected to collect a variety of enterococcus strains. For further identification, catalase, growth in Bile Esculin Agar (Oxoid), and trypticase soy broth + 6.5% NaCl tests were done. If there was any doubt about the identification, the API STREP (BioMérieux, France) was used.

### Identification of *Enterococcus* species and Cu tolerance genes

*Enterococcus* spp., *E. faecalis*, *E. faecium*, and Cu tolerance genes were determined by real-time PCR (LightCycler, Roche Diagnostics, Germany) on crude bacterial DNA (Table 1).

**Table 1:** Primers and PCR conditions used in identification of *Enterococcus* species and amplification of Cu tolerance genes

Species/Cu tolerance genes	Primer sequence	PCR conditions	Amplicon size (bp)	Reference
*Enterococcus* spp*.*	TCA ACC GGG GAG GGT	95 °C, 10 s; 60 °C, 5 s; 72 °C, 29 s	733	(Igbinosa and Beshiru 2019)
	ATT ACT AGC GAT TCC GG			
*E. faecalis*	TCA AGT ACA GTT AGT CTT TAT TAG	95 °C, 10 s; 44 °C, 5 s; 72 °C, 38 s	941	(Igbinosa and Beshiru 2019)
	ACG ATT CAA AGC TAA CTG AAT CAG T			
*E. faecium*	TTG AGG CAG ACC AGA TTG ACG	95 °C, 10 s; 55 °C, 5 s; 72 °C, 26 s	658	(Igbinosa and Beshiru 2019)
	TAT GAC AGC GAC TCC GAT TCC			
*tcrB*	CAT CAC GGT AGC TTT AAG GAG ATT TTC	95 °C, 10 s; 64 °C, 5 s; 72 °C, 27 s	663	(Hasman et al. 2006)
	ATA GAG GAC TCC GCC ACC ATT G			
*copB*	GAT TGA GCA ATC GGG TGA GT	95 °C, 10 s; 54 °C, 5 s; 72 °C, 49 s	1219	(Silveira et al. 2014)
	TGA TGG ATA TGG AGC ACG AA			

PCR was performed in a volume of 20 µL containing 4.0 µL of LightCycler FastStart DNA MasterPLUS SYBR Green I® (Roche Diagnostics, Mannheim, Germany). An initial denaturation cycle at 95 °C for 10 min was performed in all cases followed by 45 amplification cycles. Table 1 shows the denaturation, annealing, and extension conditions for each set of primers. Melting curves were plotted automatically and analyzed (LightCycler software). PCR products were checked on 2% agarose gels stained with ethidium bromide and visualized with UV light. By comparing both results, it was possible to establish specific melting temperatures to identify each gene; positive and negative controls were included. To avoid errors, the procedures were separately repeated by different technicians.

### Quantification of Cu and Zn

Determinations of Cu and Zn content in piglets’ liver and kidney were executed by flame atomic absorption spectrometry (FAAS) using an air–acetylene flame, according to an internal procedure based on ISO 6869:2000 (International Organization for Standardization Animal Feeding Stuffs — Determination of the Contents of Calcium, Copper, Iron, Magnesium, Manganese, Potassium 2023) and ISO 14082:2003 (International Organization for Standardization 2003), after dry ashing. The spectrometer used was a Thermo Scientific iCE 3000 and lamps were single-element hollow cathode lamps.

After homogenization of the samples, 5 g was weighed (fresh weight) into vitrosil crucibles and dried ashed at 450 °C in a muffle furnace, under a gradual increase in temperature. The ash from each sample was dissolved in hydrochloric acid 0.6 M and diluted to the desired volume to be read within the range of the calibration curve for both Zn and Cu. The solutions of the samples under analysis were transferred to the nebulizer, where the elements were aspirated and atomized by flame action fed with acetylene and compressed air, emitting light at the corresponding wavelengths (324.8 nm for Cu and 213.9 nm for Zn). The metal contents of the samples were derived from calibration curves, which were made up of five standards prepared by diluting commercial Cu (Merck) and Zn (Merck) standard solutions with a concentration of 1000 mg/L in hydrochloric acid, as described below. The copper’s calibration curve was obtained by filling five 100-mL class A volumetric flasks with 50 to 250 µL in 50-µL steps of the copper commercial solution and subsequent topping with a 0.6 M hydrochloric acid solution. A zero point was obtained from the dilution reagent alone. For the calibration curve of Zn, the same procedure was used, except the used Zn commercial solution volumes for the five samples were 25 to 125 µL in 25-µL steps.

An analytical quality control was also carried out. To assure the accuracy and precision of the calibration curves, two additional solutions per element were prepared using different Cu and Zn standards (copper sulfate from Panreac and zinc chloride from Supelco). First, stock solutions each with a concentration of 1 g/L per metal were prepared, based on the respective salt’s molecular weight. Secondly, additional dilutions were made to have the solutions lie at the extreme of the calibration curves.

The first control was made at the beginning of the day’s sample readings and the second at the end. Tolerances between 90 and 110% were accepted. The linearity of the curves was assessed by means of their graphical representation together with the analysis of the correlation coefficient, which was greater than 0.999 in all the curves used. The samples were analyzed in duplicate, and the result obtained is the average of the two determinations.

Values of Zn in the liver were classified according to López-Alonso et al. (López-Alonso et al. 2007): deficient (9.6–25 mg/kg), marginal (25–30 mg/kg), adequate (35–90 mg/kg), high (above 200 mg/kg), and toxic (500–3100 mg/kg). Due to the gap of 90–200 mg/kg and the lack of differentiation down to the lower toxic concentration in these definitions, the “high level” is redefined to include values from 90 to 500 mg/kg.

In the same work (López-Alonso et al. 2007), also, two levels for the concentration of Zn in the kidneys were defined: adequate (15–30 mg/kg) and toxic (190–367 mg/kg). Therefore, there is a gap between 30 and 190 mg/kg and a new level named “high level” is proposed.

Values of Cu in liver were classified according the same authors (López-Alonso et al. 2007): deficient (0.3–1.02 mg/kg), marginal (4–7 mg/kg), adequate (7–25 mg/kg), high (25–200 mg/kg), and toxic (150–15,000 mg/kg).

In the same work (López-Alonso et al. 2007), also, five levels for the concentration of Cu in kidneys were defined: deficient (2–4 mg/kg), marginal (4–7 mg/kg), adequate (7–10 mg/kg), high (12–25 mg/kg), and toxic (30–1200 mg/kg).

### Antimicrobial and metal susceptibility assays

The minimum inhibitory concentrations (MIC) for each metal, defined as the lowest concentration of the metal at which no growth was observed, were determined by the agar dilution method, applying standard bacteriological methods. Metals stock solutions (CuSO_4_∙5H_2_O and ZnCl_2_) were added to melted Mueller–Hinton (MH) agar to obtain final concentrations ranging from 0.125 to 16 mM for CuSO_4_ and 0.5 to 32 mM for ZnCl_2_ (Aarestrup and Hasman 2004). Culture of each isolate was diluted to 1 × 10^7^ CFU/mL; 1 µL of this dilution was inoculated as spots with a microplate replicator, followed by overnight incubation at 37 °C. Each assay was performed in triplicate. The well-grown colonies were regarded as bacteria with resistance to the heavy metals. The plates without Cu and Zn were used as control.

The enterococcus isolates were tested for antimicrobial susceptibility by agar disk diffusion method on MH agar according to the guidelines provided by the European Committee for Antimicrobial Susceptibility Testing (EUCAST 2020), to the following antimicrobial agents (Liofilchem®s.r.l., Italy): vancomycin (VAN), ciprofloxacin (CIP), linezolid (LNZ), tigecycline (TIG), ampicillin (AMP), and imipenem (IP).

### Statistical analysis

Data is presented in terms of absolute and/or relative frequencies and associations were evaluated through the Fisher exact test. Analyses were conducted using IBM SPSS, version 27, and evaluated at a 5% significance level.

## Results

### Levels of Zn in the kidney and MICs of Zn in *Enterococcus* species

The Zn content in the kidneys of 43 Portuguese piglets was determined. Concentrations of Zn (Table 2) were within the adequate range in 67% of the piglets. The remaining 33% were above this limit, being 7% allocated to the toxic level and 26% in a “high-level” range.

**Table 2:** Number of *Enterococcus* species in Zn levels in piglets’ kidney and liver and respective Zn MICs

Piglet (*N* =)	Zn level	Enterococci (*N* =)	*Enterococcus* species (*N* =)	Zn MIC mM (*N* =)					
				32	16	8	4	2	1
43	Kidney	128							
3	Toxic	15	*E. faecalis* (11)	6	5	-	-	-	-
			*E. faecium* (3)	1	1	1	-	-	-
			*Enterococcus* spp. (1)	-	-	-	-	1	-
11	High level	27	*E. faecalis* (11)	8	1	-	-	2	-
			*E. faecium* (12)	3	3	5	1	-	-
			*Enterococcus* spp. (4)	1	-	-	3	-	-
29	Adequate	86	*E. faecalis* (18)	5	5	2	3	2	1
			*E. faecium* (30)	2	5	19	4	-	-
			*Enterococcus* spp. (38)	1	6	4	22	5	-
42	Liver	121							
			*E. faecalis* (18)	12	6	-	-	-	-
8	Toxic	31	*E. faecium* (11)	4	4	3	-	-	-
			*Enterococcus* spp. (2)	-	-	-	1	1	-
19	High level	52	*E. faecalis* (11)	4	2	2	-	3	-
			*E. faecium* (23)	2	3	16	2	-	-
			*Enterococcus* spp. (18)	2	5	1	10	-	-
			*E. faecalis* (8)	3	3	-	-	1	1
15	Adequate	38	*E. faecium* (11)	-	2	6	3	-	-
			*Enterococcus* spp. (19)	-	1	3	10	5	-

**Fig. 1:**
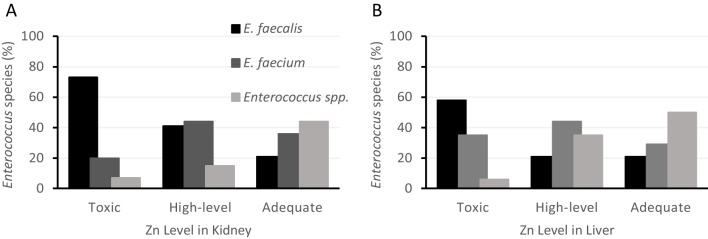
Distribution of *Enterococcus* species by the Zn levels in kidney (**A**) and liver (**B**) of piglets

From the feces of these 43 piglets, 128 enterococci were isolated and identified as a biomarker of piglets’ intestinal microbiota: 40 *E. faecalis*, 45 *E. faecium*, and 43 *Enterococcus* spp. The distribution of *Enterococcus* species among the different levels of Zn concentrations in the kidneys can be observed in Fig. 1A, and no statistically significant association is observed (Fisher’s exact test: *p* = 0.359). The percentage of *E. faecalis* was maximum at the toxic level (73%), decreased in the “high level” (41%), and reached the minimum at the adequate level (21%). The percentage of *E. faecium* was the lowest at the toxic level (20%) and the highest at the “high level” (44%), reaching an intermediate value at the adequate level (36%). For *Enterococcus* spp., the minimum percentage was observed in the toxic level (7%), increasing in the “high level” (15%), and reaching the maximum in the adequate level (44%).

In piglets with Zn adequate level in the kidney, 86 enterococci were isolated: 18 (21%) *E. faecalis*, 30 (36%) *E. faeci*um, and 38 (44%) *Enterococcus* spp*.* (Table 2)*.* The Zn MICs were determined and range from 1 to 32 mM. In the literature, there is no agreement about the cut-offs of Zn MICs in species of enterococci (Aarestrup and Hasman 2004; Mazaheri Nezhad Fard et al. 2011); therefore, regarding the results in this study, the cut-off of 16 mM to Zn was adopted, considering that the isolates with reduced susceptibility to Zn had a Zn MIC ≥ 16 mM. In *Enterococcus* spp., the MIC of 4 mM was predominant (58%). The MICs of *E. faecalis* were distributed over the concentrations tested, with 50% above 16 mM. For *E. faecium*, the most determined MIC was 8 mM (63%) (Table 2).

In the “high level,” 27 enterococci were isolated: 11 (41%) *E. faecalis*, 12 (44%) *E. faecium*, and four (15%) *Enterococcus* spp. MICs in *E. faecalis* were mostly 32 mM (73%). Regarding *E. faecium*, 50% of the isolates had MICs of 32 mM and 16 mM. The other species of enterococci showed MICs of 32 and 4 mM (Table 2).

In the toxic level, 15 enterococci were isolated: 11 (73%) *E. faecalis*, three (20%) *E. faecium*, and one (7%) *Enterococcus* sp. All the *E. faecalis* had MICs of 32 mM (55%) and 16 mM (45%); the three *E. faecium* had MICs of 32 mM, 16 mM, and 8 mM, and those in *Enterococcus* spp. were 2 mM (Table 2).

### Levels of Zn in the liver and MICs of Zn in *Enterococcus* species

The content of Zn in the liver was determined in 42 piglets. Concentrations of Zn (Table 2) were within the adequate range in 36% of the piglets. The remaining 64% were above this limit, being 45% allocated in “high level” and 19% in the toxic level. There were no piglets with deficient or marginal level of Zn in the liver.

From feces of these 42 piglets, 121 enterococci were isolated and identified: 37 *E. faecalis*, 45 *E. faecium*, and 39 *Enterococcus* spp. The distribution of enterococci species among the different levels of Zn in the liver can be observed in Fig. 1B. Zn levels in piglet’s liver were associated with *Enterococcus* species (Fisher’s exact test: *p* < 0.001). The percentage of *E. faecalis* was maximum at the toxic level (58%) and decreased in the “high level” and adequate level (21%). The percentage of *E. faecium* was the lowest (29%) at the adequate level and the highest (44%) at the “high level,” reaching an intermediate value (35%) at the toxic level. For *Enterococcus* spp., the minimum percentage (6%) was observed in the toxic level, increasing to 35% in the “high level” and achieving the maximum (50%) in the adequate level.

In the piglets with Zn adequate level in the liver, 38 enterococci were isolated: eight (21%) *E. faecalis*, 11 (29%) *E. faecium*, and 19 (50%) *Enterococcus* spp. (Table 2). The Zn MICs in *E. faecalis* were 32 mM, 16 mM, 2 mM, and 1 mM; in *E. faecium* ranged from 16 to 4 mM; and in *Enterococcus* spp. ranged from 16 to 2 mM (Table 2). In the “high level,” 52 enterococci were isolated: 11 (21%) *E. faecalis*, 23 (44%) *E. faecium*, and 18 (35%) *Enterococcus* spp. The Zn MICs in *E. faecalis* were 32 mM, 16 mM, 8 mM, and 2 mM; in *E. faecium*; and in *Enterococcus* spp. ranged from 32 to 4 mM (Table 2). In the toxic level were isolated 31 enterococci: 18 (58%) *E. faecalis*, 11 (35%) *E. faecium*, and 2 (6%) *Enterococcus* spp. The Zn MICs in *E. faecalis* were 32 mM and 16 mM; in *E. faecium* ranged from 32 to 8 mM; and in *Enterococcus* spp. were 4 mM and 2 mM.

### MICs of Zn and antimicrobial resistance of *Enterococcus* species

Antibiotic susceptibility of enterococci was determined and considering the Zn MICs ≥ 16 mM, 53 enterococci were found (30 *E. faecalis*, 15 *E. faecium*, and 8 *Enterococcus* spp.). Full susceptibility to the antibiotics tested was observed in 27% of these enterococci, and 73% had at least one resistance to antibiotics (Table 3). In this group, three enterococci were resistant to three antibiotics among the six tested. They were resistant to VAN and CIP, and the third antibiotic was AMP in *E. faecalis*, IP in *E. faecium*, and LNZ in *Enterococcus* spp. The resistance to IP and CIP was similar. No statistically significant association was found between the *Enterococcus* species and antimicrobial resistance (Fisher’s exact test: *p* = 0.082).

**Table 3:** Relation between Zn MIC and antimicrobial resistance of *Enterococcus* species

Zn MIC (mM)	Enterococci (*N* = 128)	*Enterococcus* species	Antimicrobial resistance (*N* =)						Full susceptibility (%)
		(*N* =)	VAN	CIP	LNZ	TIG	AMP	IP	
32, 16	53	*E. faecalis* (30)	1	15	1	-	1	6	27
		*E. faecium* (15)	1	4	-	-	1	10	
		*Enterococcus* spp. (8)	1	2	1	-	-	5	
8, 4, 2, 1	75	*E. faecalis* (10)	-	3	-	-	-	-	48
		*E. faecium* (30)	-	3	-	-	1	19	
		*Enterococcus* spp. (35)	2	15	-	-	1	1	

- not detected

Seventy-five enterococci (10 *E. faecalis*, 30 *E. faecium*, and 35 *Enterococcus* spp.) had Zn MICs < 16 mM. Full susceptibility was observed in 48% of the enterococci. All the isolates were susceptible to LNZ and TIG; 52% presented resistance to at least one of the four antibiotics. Resistance to CIP and IP were similar and higher relatively to the other antibiotics. Three *E. faecalis* isolates only presented resistance to CIP. Nineteen isolates of *E. faecium* were resistant to IP and three to CIP. *Enterococcus* spp. presented 15 isolates resistant to CIP and 2 to VAN. The *Enterococcus* species seems to be statistically associated with antimicrobial resistance (Fisher’s exact test: *p* < 0.001) (Table 3).

### Levels of Cu in the kidney and MICs of Cu in *Enterococcus* species

The content of Cu in the kidneys was determined in 43 piglets. Concentrations of Cu (Table 4) were within the adequate range in 12% of the piglets; 58% were below this limit, being 35% allocated to the marginal level and 23% to the deficient level. The other 31% were distributed in the high (12%) and toxic levels (19%).

**Table 4:** Number of *Enterococcus* species in Cu levels in piglets’ kidney and liver and respective Cu MICs

Piglet (*N* =)	Cu level	Enterococci (*N* =)	*Enterococcus* species (*N* =)	Cu MIC mM (*N* =)				
				8	4	2	1	0.25
43	Kidney	128						
8	Toxic	31	*E. faecalis* (18)	14	4	-	-	-
			*E. faecium* (11)	10	1	-	-	-
			*Enterococcus* spp. (2)	-	2	-	-	-
5	High	7	*E. faecalis* (2)	-	1	1	-	-
			*E. faecium* (3)	-	2	1	-	-
			*Enterococcus* spp. (2)	-	-	2	-	-
5	Adequate	15	*E. faecalis* (4)	1	1	2	-	-
			*E. faecium* (4)	2	-	2	-	-
			*Enterococcus* spp. (7)	1	3	3	-	-
15	Marginal	45	*E. faecalis* (10)	-	5	5	-	-
			*E. faecium* (17)	12	3	1	-	1
			*Enterococcus* spp. (18)	4	7	7	-	-
10	Deficient	30	*E. faecalis* (6)	2	3	1	-	-
			*E. faecium* (10)	6	3	1	-	-
			*Enterococcus* spp. (14)	3	7	1	3	-
42	Liver	121						
			*E. faecalis* (6)	-	3	3	-	-
6	High	18	*E. faecium* (3)	-	-	3	-	-
			*Enterococcus* spp. (9)	2	3	4	-	-
			*E. faecalis* (16)	9	3	4	-	-
20	Adequate	55	*E. faecium* (22)	14	7	1	-	-
			*Enterococcus* spp. (17)	6	3	5	3	-
			*E. faecalis* (3)	1	1	1	-	-
9	Marginal	26	*E. faecium* (12)	10	1	-	-	1
			*Enterococcus* spp. (11)	-	7	4	-	-
			*E. faecalis* (12)	7	4	1	-	-
7	Deficient	22	*E. faecium* (8)	6	2	-	-	-
			*Enterococcus* spp. (2)	-	2	-	-	-

**Fig. 2:**
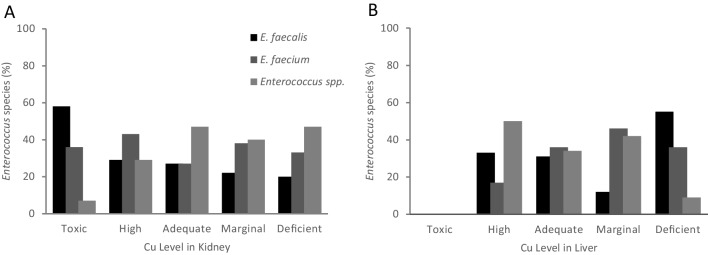
Distribution of *Enterococcus* species by the Cu levels in kidney (**A**) and liver (**B**) of piglets

The distribution of enterococci species (*N* = 128) among the different levels of Cu in the kidney could be observed in Fig. 2A. The percentage of *E. faecalis* was maximum (58%) at the toxic level, decreasing in the other levels and reaching the minimum (20%) at the deficient level. The percentage of *E. faecium* varied from 43% in the high level to 27% in the adequate level. For *Enterococcus* spp., the minimum percentage (7%) was observed in the toxic level, rising to 47% in the adequate and deficient levels. Cu levels in piglet’s kidney were associated with the detected *Enterococcus* species (Fisher’s exact test: *p* = 0.005).

In the piglets with Cu adequate level in the kidney, 15 enterococci were isolated: four (27%) *E. faecalis*, four (27%) *E. faecium*, and seven (47%) *Enterococcus* spp. As there is no consensus on the cut-off of Cu MIC for *Enterococcus* species in the literature (Aarestrup and Hasman 2004; Mazaheri Nezhad Fard et al. 2011; Mourão et al. 2016), in this study, a cut-off of 8 mM to Cu was adopted, considering that the isolates with reduced susceptibility to Cu had a Cu MIC ≥ 8 mM. The Cu MIC ranged in *E. faecalis* and *Enterococcus* spp. from 8 to 2 mM; the Cu MICs in *E. faecium* were 8 and 2 mM (Table 4).

In the piglets with Cu high level in the kidney, seven enterococci were isolated: two (29%) *E. faecalis*, three (43%) *E. faecium*, and two (29%) *Enterococcus* spp. The Cu MICs in *E. faecalis* and in *E. faecium* were 4 mM and 2 mM; in *Enterococcus* spp. were 2 mM (Table 4). In the piglets with toxic level, 31 enterococci were isolated: 18 (58%) *E. faecalis*, 11 (35%) *E. faecium*, and two (7%) *Enterococcus* spp. The MIC in *E. faecalis* and in *E. faecium* was 8 mM and 4 mM; and in *Enterococcus* spp. was 4 mM (Table 4).

For the piglets displaying a Cu marginal level in the kidney, 45 enterococci were isolated: 10 (22%) *E. faecalis*, 17 (38%) *E. faecium*, and 18 (40%) *Enterococcus* spp. The MICs in *E. faecalis* were 4 mM and 2 mM; in *E. faecium* were 8 mM, 4 mM, 2mM, and 0.25 mM; and in *Enterococcus* spp. ranged from 8 to 2 mM (Table 4). In the piglets with a Cu deficient level, 30 enterococci were isolated: six (20%) *E. faecalis*, 10 (33%) *E. faecium*, and 14 (47%) *Enterococcus* spp. The MIC in *E. faecalis* and in *E. faecium* ranged from 8 to 2 mM; and in *Enterococcus* spp. ranged from 8 to 1 mM (Table 4).

### Levels of Cu in the liver and MICs of Cu in *Enterococcus* species

The content of Cu in the liver was determined in 42 piglets. Concentrations of Cu (Table 4) were within the adequate level in 48% of these animals. Thirty-eight percent were below the adequate limit, being 21% allocated in the marginal level and 17% in deficient level. The others 14% were in high level, and the toxic level was not detected.

The distribution of enterococci species among the different levels of Cu in the liver could be observed in Fig. 2B. The percentage of *E. faecalis* was maximum (55%) at the deficient level, decreasing in the other levels and reaching the minimum (12%) at the marginal level. The percentage of *E. faecium* varied from 46% in marginal level to 17% in the high level. For *Enterococcus* spp., the maximum percentage (50%) was observed in the high level, and minimum in the deficient level (9%). Cu levels in piglet’s liver were statistically associated with *Enterococcus* species (Fisher’s exact test: *p* = 0.007).

In the piglets with Cu adequate level in the liver, 55 enterococci were isolated: 16 (29%) *E. faecalis*, 22 (40%) *E. faecium*, and 17 (31%) *Enterococcus* spp. The Cu MICs ranged in *E. faecalis* and *E. faecium* from 8 to 2 mM; and in *Enterococcus* spp. from 8 to 1 mM (Table 4). In the piglets with Cu high level in the livers, 18 enterococci were isolated: six (33%) *E. faecalis*, three (17%) *E. faecium*, and nine (50%) *Enterococcus* spp. The MICs in *E. faecalis* were 4 mM and 2 mM; in *E. faecium* were 2 mM; and in *Enterococcus* spp. ranged from 8 to 2 mM (Table 4).

In the piglets with Cu marginal level in the liver, 26 enterococci were isolated: three (12%) *E. faecalis*, 12 (46%) *E. faecium*, and 11 (42%) *Enterococcus* spp. The Cu MICs in *E. faecalis* ranged from 8 to 2 mM; in *E. faecium* were 8, 4, and 0.25 mM; and in *Enterococcus* spp. were 4 mM and 2 mM (Table 4). In the piglets with Cu deficient level in the liver, 22 enterococci were isolated: 12 (55%) *E. faecalis*, 8 (36%) *E. faecium*, and 2 (9%) *Enterococcus* spp. The Cu MICs in *E. faecalis* ranged from 8 to 2 mM, in *E. faecium* 8 mM and 4 mM, and in *Enterococcus* spp. 4 mM (Table 4).

### MICs and tolerance genes of Cu and antimicrobial resistance of *Enterococcus* species

The antibiotic susceptibility of enterococci was determined and, considering the Cu MICs ≥ 8 mM, 55 enterococci were included (17 *E. faecalis*, 30 *E. faecium*, and 8 *Enterococcus* spp.). Twenty-two percent of the enterococci were susceptible to all the antibiotics tested. The other 78% had resistance to at least one antibiotic and were susceptible to TIG (Table 5). In this group, one *E. faecium* was resistant to three antibiotics (VAN, CIP, and IP). The resistance to IP and CIP was the highest, being IP resistance the most observed. VAN resistance was observed in two *Enterococcus* spp. and in one *E. faecium*. Eleven *E. faecalis*, four *E. faecium*, and two *Enterococcus* spp. isolates demonstrated resistance to CIP. IP resistance was mostly detected in *E. faecium* (24 isolates), but two *E. faecalis* and three *Enterococcus* spp. showed also this resistance. The *Enterococcus* species were statistically associated with antimicrobial resistance (Fisher’s exact test: *p* < 0.001).

**Table 5:** Relation between Cu MIC and antimicrobial resistance of *Enterococcus* species

Cu MIC (mM)	Enterococci (*N* = 128)	*Enterococcus* species (*N* =)	Cu tolerance genes (*N* =)			Antimicrobial resistance (*N* =)						Full susceptibility (%)
			*tcr*B	*cop*B	*trc*B + *cop*B	VAN	CIP	LNZ	TIG	AMP	IP	
8	55	*E. faecalis* (17)	13	-	2	-	11	1	-	-	2	22
		*E. faecium* (30)	6	9	12	1	4	-	-	2	24	
		*Enterococcus* spp. (8)	4	1	-	2	2	-	-	1	3	
4, 2, 1, 0.25	73	*E. faecalis* (23)	4	2	-	1	7	-	-	1	4	53
		*E. faecium* (15)	3	2	-	-	3	-	-	-	5	
		*Enterococcus* spp. (35)	2	8	-	1	15	1	-	-	3	

- not detected

Seventy-three enterococci (23 *E. faecalis*, 15 *E. faecium*, and 35 *Enterococcus* spp.) had Cu MICs < 8 mM (susceptible to Cu). Full susceptibility was observed in 53% enterococci. All the isolates were susceptible to TIG, and 47% presented resistance to at least one of the other five antibiotics. Resistance to CIP was the most detected followed by IP (Table 5). The *Enterococcus* species were statistically independent from antimicrobial resistance (Fisher’s exact test: *p* = 0.188).

Among the 55 enterococci with Cu MIC ≥ 8 mM, the *tcr*B gene, involved in reduced susceptibility to Cu, was detected in 13 of the 17 *E. faecalis*, six of the 30 *E. faecium*, and four of the eight *Enterococcus* spp. The *cop*B gene was observed in nine *E. faecium* and in one *Enterococcus* spp. In two *E. faecalis* and 12 *E. faecium*, both genes were observed (Table 5). Highlighting that the presence of the genes was consistent, noting that 15 of the 17 *E. faecalis* had *tcr*B or *tcr*B + *cop*B; 27 of the 30 *E. faecium* had *tcr*B, *cop*B, or *tcr*B + *cop*B, and in eight *Enterococcus* spp., five presented *tcr*B or *cop*B.

In the other 73 enterococci with Cu MIC < 8 mM, four *tcr*B genes were detected in 23 *E. faecalis*, three in 15 *E. faecium*, and two in 35 *Enterococcus* spp. The *cop*B gene was observed in two *E. faecalis*, two *E. faecium*, and eight *Enterococcus* spp. (Table 5). In this group, the presence of genes was much lower than in the former, and the association of two genes in the same isolate was not observed.

## Discussion

Particularly in pig production, heavy-metal-containing compounds for growth promotion and therapy of intestinal diseases are used at inhibitory rather than lethal concentrations, although the overuse of these ions may conduct to toxicity in animals and later in human health and environmental contamination with negative impact in the “One Health” perspective (Poole 2017; Rensing et al. 2018).

The sampling used in this work was random and blind, as the Portuguese reality regarding the use of Zn and Cu is unknown and this study was the first to be carried out in this context. Therefore, several samples were collected from different locations in Portugal and there was no access to the data (feed, additives, therapies, etc.) how pig farmers raised the piglets, and this may be a limitation of the study.

The concentrations of Zn and Cu in the kidney and liver of slaughtered piglets were determined in order to observe if their limits were overcome or not in swine production. In Portugal, as well as in other countries, the consumption of piglets’ viscera is huge, being a gastronomic delicacy, and therefore, the concern about the levels of these metallic ions in these ingredients. The excessive consumption of Zn and Cu could lead to anemia and immunologic alterations both in animals and in humans (Rensing et al. 2018). The obtained results of Zn and Cu concentrations in tissues were organized according to López-Alonso et al. (López-Alonso et al. 2007). However, in those definitions, between the “adequate” and “toxic-level” concentrations of Zn in the kidney, there are no other categorizations. As 26% of the piglets were within this gap, a new level designated as “high level” is proposed. Concerning Zn amounts in the liver, there is also a gap between adequate and high. The 24% piglets included in this gap were joined with the piglets in the high level (21%), in order to better define the results obtained above the adequate and below toxic level. Thus, the insert of another level is proposed named “high level,” the same designation used in Zn kidney concentrations.

In a study of López-Alonso et al. (López-Alonso et al. 2007) of intensive farming in Galicia, all animals had concentrations of Cu and Zn in the adequate levels. However, in the present study, a significant number of piglets with Zn and Cu level concentrations above those deemed adequate were observed. Zn toxic level was found in 19% of the piglet’s livers and 7% of the kidneys. Also, 19% of piglets had a Cu toxic level in the kidney and 14% had a high level in the liver. Other authors had already described Zn toxic hepatic level in piglets (Gabrielson et al. 1996).

In this study, the correlation between Zn and Cu concentrations in piglets’ kidney and liver and species of enterococci isolated from fecal microbiota was done. A statistical association was observed between the Zn concentration in the liver, Cu in the kidney and liver, and the enterococci species. *E. faecalis* was prevalent in the liver and kidney displaying a Zn and Cu toxic level and *E. faecium* in Zn and Cu high levels, and *Enterococcus* spp. were predominant in adequate and below levels. This study is innovative, because there is no data available in the literature that allow the comparison of these results. Several studies relate the behavior of fecal enterococci with the concentrations of Cu and Zn present in the pigs’ diet (Aarestrup and Hasman 2004; Mazaheri Nezhad Fard et al. 2011; Wales and Davies 2015; Capps et al. 2020) while this work relates the bacteria with the levels of Zn and Cu in the liver and kidney.

The highest Zn MICs were observed in enterococci species isolated from piglets with a Zn toxic level in the kidney and liver, followed by the isolates from the “high level.” The isolates allocated in the adequate level of Zn had the lowest MICs. According to these results, it appeared that the introduction of the “high level” is essential to fill the existing gap.

Independently of the Zn levels in the kidney and liver of piglets, the *E. faecalis* showed the greatest reduced susceptibility to Zn (MIC ≥ 16 mM) like other authors (Mazaheri Nezhad Fard et al. 2011). Regardless of the Cu levels in the kidney and liver of piglets, *E. faecium* showed the greatest reduced susceptibility to Cu (MIC of 8 mM) similarly to other authors results (FEEDAP 2016).

In this study, among the isolates with a Zn MIC ≥ 16 mM, 73% had at least one resistance to the antibiotics tested. Twenty-one isolates were resistant to CIP and other 21 were resistant to IP. These antibiotics are widely used in human clinical therapy (Mazaheri Nezhad Fard et al. 2011). Resistance to CIP occurs, most of the time, due to the chromosomal mutations in the genes that encode DNA gyrase and topoisomerase IV (Drlica et al. 2008), but the chromosomic or plasmid-borne quinolone resistance gene *qnr* is also worth considering (Jacoby et al. 2008). Resistance to IP, was particularly expressed by *E. faecium* due to alteration or hyperproduction of PBPs, and this expression could be located in the chromosome or in plasmids (Hollenbeck and Rice 2012). Cu, Zn, and antibiotic resistance genes co-occur in animal isolates in plasmids or genomic islands, and this genetic linkage could explain the potential for metals to drive antibiotic resistance development in human pathogens (Poole 2017).

In general, the isolates with reduced susceptibility to Zn or Cu presented more resistances to antibiotics, than the isolates considered susceptible to Zn and Cu. Reduced susceptibility to Cu in enterococci has been shown to be plasmid encoded and is often associated with resistance to antibiotics such as macrolides and glycopeptides (Rensing et al. 2018). Some authors demonstrated that Cu and Zn at high levels provide selective pressure for metal resistance, which, in turn, can drive the resistance to antibiotics owing to genetic and physiological linkages between the two (Knapp et al. 2011; Poole 2017; Rensing et al. 2018).

The Cu reduced susceptibility is usually mediated by a plasmid-borne *tcr*B gene that has been initially reported in pig *E. faecium* isolates and later in various enterococci species (Hasman et al. 2006). In this study, this gene was observed in 36% of enterococci isolates. This result is similar to that reported in a Danish study (Hasman et al. 2006), inferior to an Australian one (Mazaheri Nezhad Fard et al. 2011) and superior to those reported in investigations done in the USA (Capps et al. 2020). Sixty-seven percent of isolates with a Cu MIC of 8 mM were *tcr*B positive, while only 12% were *tcr*B positive within the isolates with a MIC < 8 mM. These results were similar to those of the USA (Capps et al. 2020). Another gene associated with Cu tolerance is *cop*B, homologous to *tcr*B, and its presence was similar in enterococci isolates. Both genes were detected in 14 isolates (Cu MIC 8 mM), highlighting that the majority of the enterococci with reduced susceptibility to Cu presented the Cu tolerance genes alone or in combination and were also more resistant to the tested antibiotics, suggesting a possible linkage between the Cu and antibiotic resistances (Knapp et al. 2011; Poole 2017; Rensing et al. 2018).

## Conclusions

This innovative study showcases the relationship between the levels of Zn and Cu in the piglets’ kidney and liver and the decreased susceptibility to Zn and Cu and to antibiotics by enterococci. Enterococci from piglets with Zn and Cu high or toxic levels displayed a greater tolerance to these ions, reinforced by the presence of Cu tolerance genes and also by resistance to antibiotics. These findings were not yet reported by other authors, although various studies stated the relationship between the high use of metals in feeds and reduced susceptibility to ions and antibiotics in enterococci (Hasman et al. 2006; Yazdankhah et al. 2014; Agga et al. 2015; Wales and Davies 2015; Capps et al. 2020).

From the obtained results, it could be hypothesized that the piglets’ feed was being supplemented with Zn and Cu in quantities higher than allowed by European legislation, according to the Commission Regulation (EC) No. 1334/2003 (European Commission 2003). In addition, these high levels of Zn and Cu appear to select more resistant bacteria to both antibiotics and metals, which in turn might contaminate the environment and spread to humans. Such information can help in the implementation of measures and new policies aligned with “One Health.”

## Data Availability

All the data and tools/models used for this work are publicly available.
